# Effect of pregnancy induced hypertension on adverse perinatal outcomes in Tigray regional state, Ethiopia: a prospective cohort study

**DOI:** 10.1186/s12884-019-2708-6

**Published:** 2019-12-31

**Authors:** Abadi Kidanemariam Berhe, Abiodun O. Ilesanmi, Christopher O. Aimakhu, Afework Mulugeta

**Affiliations:** 1 College of Medicine and Health Sciences, Adigrat University, Tigray, Ethiopia; 2 Pan African University Institute for Life and Earth Sciences, University of Ibadan, Ibadan, Nigeria; 3 Department of Obstetrics and Gynaecology, College of Medicine, University College Hospital, University of Ibadan, Ibadan, Nigeria; 4 School of Public Health, College of Health Sciences, Mekelle University, Tigray, Ethiopia

**Keywords:** Adverse perinatal outcomes, Pregnancy-induced hypertension, Tigray, Ethiopia

## Abstract

**Background:**

The prevalence of pregnancy-induced hypertension in Ethiopia ranges from 2.2 to 18.3%. However, so far little is known about the adverse perinatal outcomes of pregnancy-induced hypertension in Tigray regional state, Ethiopia. Therefore, the objective of this study was to assess the effect of pregnancy-induced hypertension on adverse perinatal outcomes in Tigray Regional State, Ethiopia.

**Methods:**

a prospective cohort study was conducted on a total sample of 782 pregnant women attending antenatal care in hospitals of Tigray regional state, Ethiopia. Pregnant mothers diagnosed with PIH during the data collection period in the selected hospitals were included as exposed group and normotensive women were also enrolled as a control group. This study addresses women diagnosed with preeclampsia, eclampsia and gestational hypertension between 28 and 35 weeks of gestation. Data were collected using an interviewer-administered questionnaire and review of their medical records from February 2018, to February 2019. The adverse perinatal outcome event includes low birth weight, birth asphyxia, small for gestational age, preterm delivery, admission to neonatal intensive care unit and perinatal death. A modified Poisson regression model with robust standard errors was used to analyze relative risk.

**Results:**

In this study, the overall incidence of adverse perinatal outcome was higher among women with pregnancy-induced hypertension than normotensive women (66.4% vs 22.2%). After adjusted for confounders women with pregnancy-induced hypertension were born babies with a higher risk of low birth weight (adjusted RR (95%CI) = 5.1(3.4,7.8)), birth asphyxia (aRR = 2.6(1.9,3.8)), small for gestational age (aRR = 3.3(2.3,4.6)), preterm delivery (aRR = 5.2(3.4,7.9)), stillbirth (aRR = 3.46(1.40,8.54)), admission to neonatal intensive care unit (aRR = 5.1(3.1,8.4)) and perinatal death (aRR = 3.6(1.8,7.4)) compared to normotensive pregnant women.

**Conclusions:**

Higher incidences of adverse perinatal outcomes occurred among women pregnancy-induced hypertension in Tigray regional state, Ethiopia. Hence, health care providers should strengthen prevention, early diagnosis and prompt management of pregnancy-induced hypertension to reduce adverse perinatal outcomes of pregnancy-induced hypertension.

## Introduction

Pregnancy-induced hypertension (PIH) was defined as new hypertension that appears at 20 weeks or more gestational age of pregnancy with or without proteinuria, which includes gestational hypertension, pre-eclampsia, and eclampsia [1–3]. Hypertension is defined as a sustained systolic BP ≥140 mmHg or diastolic BP ≥ 90 mmHg based on the average of at least two measurements, using the same arm [4]. Globally, PIH is a significant public health threat both in developed and developing countries contributing to high perinatal deaths [5]. PIH complicates 2–8% of pregnancies in the Western world [6]. However, the magnitude of PIH in developing countries reaches up to 16.7% [7]. Additionally, the available literature in Ethiopia showed a high burden of PIH which ranges from 2.23 to 18.25% [8–13]. Similarly, according to the finding of a study conducted in Tigray regional state, PIH was among the leading obstetric causes of maternal mortality in the region and the prevalence of PIH reported in this study was 8.1%, this was higher than the national pooled prevalence of PIH (6.29%) [14–16].

Different studies conducted in developed and developing countries on adverse perinatal outcomes of pregnancy-induced hypertension showed that PIH was associated with higher rates of morbidity and mortality such as preterm delivery, low birth weight, birth asphyxia, stillbirth and early neonatal death [17–22]. Similarly, studies conducted in Africa revealed that adverse perinatal outcomes such as perinatal death, low birth weight, preterm birth, and birth asphyxia were associated with PIH [23–25]. However, the risk and incidence of adverse perinatal outcomes of PIH vary across countries, populations and ethnic-geographic areas.

In Ethiopia, despite the inconsistent findings on the incidence of adverse perinatal outcomes across the studies, the available limited studies revealed a higher incidence of low birth weight, stillbirth, early neonatal death, birth asphyxia and preterm birth among women with PIH [26, 27]. However, so far little is known about the adverse perinatal outcomes of PIH in Tigray regional state. The available studies conducted in Ethiopia were descriptive they did not address the association between PIH and adverse perinatal outcomes. Additionally, most of the studies conducted in Ethiopia were used retrospective study design which might have introduced bias due to incompleteness and misclassification of the data at the health facilities. Thus, this prospective cohort study helps to explore the effect of PIH on the adverse perinatal outcomes and reduces the bias-related with incompleteness and misclassification of the data.

## Methods

### Study setting, design and period

A prospective cohort study was conducted in multiple hospitals of Tigray regional state. Tigray regional state is located in the northern part of Ethiopia bordered by Eritrea to the north, Sudan to the west, Afar region to the east and Amhara region to the south. The source population for this study was all pregnant women who attend antenatal care at general hospitals located in Tigray from February 2018 to February 2019. General hospitals provide outpatient and inpatient services to the general population which includes medical, surgical, pediatric, accident and emergency services, maternal and child health (MCH), and obstetric and gynecological care, and other relevant services. In addition, general hospitals serve as a referral center to primary health care units. There are fifteen general hospitals in Tigray regional state from those the following eight hospitals entirely distributed in the region namely Lemlem Carl, Mekelle, Adigrat, Adwa, Saint Marry Axum, Suhul Shire, and Kahsay Abera hospitals were included in this study. Those selected hospitals can represent the larger region since more than 50 % of the hospitals in the region were included in this study. General hospitals provide both basic and comprehensive emergency obstetric and newborn care. The average number of delivery in each hospital is around 1600 per year. All the hospitals included in this study provide comprehensive diagnostic and management services for hypertensive disorders of pregnancy starting from the mild form of gestational hypertension to the severe forms of preeclampsia/eclampsia.

### Inclusion and exclusion criteria

Women with PIH and normotensive women in each antenatal care clinic of the hospitals were enrolled to study by reviewing their blood pressure level and proteinuria. Pregnancy-induced hypertension was defined as new hypertension (systolic BP ≥140 mmHg and/or diastolic BP ≥ 90 mmHg) that appears at 20 weeks or more gestational age of pregnancy with or without proteinuria (includes preeclampsia, eclampsia, and gestational hypertension). Pregnant mothers diagnosed with PIH during the data collection period in the selected antenatal care clinic of the hospitals were included as exposed participants and women without PIH during the same period were also enrolled as a non-exposed participant. Pregnant women with chronic hypertension, critically ill women who could not give consent, women who could not respond to the interview and those pregnant women likely to become “lost” e.g., planning to move, unwilling to return for the prospective follow up period were excluded from this study at the time of enrollment. There were no other restrictions (like maternal age, singleton pregnancies, etc.).

### Sample size determination and sampling procedure

A double population proportion formula was used to calculate the sample size. The maximum sample for this study was calculated from the outcome variable stillbirth by considering the following assumptions; two-sided confidence level of 95%, the power level = 80%, r = the ratio of exposed to unexposed group 1 to 2, p_1_ **=** proportion of stillbirth among women with PIH 5.4% [23], p2 **=** proportion of stillbirth among normotensive pregnant women 1.3%) [23]. With the consideration of a 10% loss to follow up a total of 798 study participants (266 participants with PIH and 532 normotensive participants) were finally included in this study.

From a total of fifteen general hospitals providing maternal health services like antenatal care, delivery and postnatal care services in Tigray regional state, randomly eight hospitals were selected using a simple random sampling technique. The calculated sample size was proportionally allocated to selected hospitals based on the number of pregnant mothers attending antenatal care in each hospital. Women with PIH were recruited consecutively until we get the required sample and two normotensive women next to diagnosed PIH cases were selected by a systematic sampling method using antenatal care registration as a frame list.

### Data collection instruments and quality assurance

A structured questionnaire containing information on socio-demographic characteristics (maternal age, residence, educational status, and employment), obstetric and reproductive health history information’s like gravidity, prior history of PIH, maternal undernutrition ((MUAC< 23 cm), history of anemia, current PIH status, and perinatal outcomes were used to collect the data. Wealth index of participants was assessed using socioeconomic variables like durable asset ownership, ownership of housing, type of floor and roof materials, ownership of farmland, farm animals, number of people in a household, number of rooms in the household, access to utilities and infrastructure (sanitation facility and source of water supply). The questionnaire was prepared by reviewing research articles, demographic health survey (DHS) tools, WHO survey tools and published works on PIH then adapted to the local context [21, 23, 27–36]. Overall the questionnaire of this study was initially prepared in English version and translated to the local language called Tigrigna by a language expert and back converted again to English by another person to check the consistency.

Midwives and nurses having experience in research assistance were involved in the data collection and supervision. Training was provided for all data collectors and supervisors before the commencement of the actual data collection. The training focused on the objectives of the study, ethical issues, interviewing techniques, inclusion criteria of the study, follow up procedures of the study and overall contents of the data collection instrument. Before starting the actual data collection, a pretest was done for the data collection instruments. Necessary corrections on the data collection instrument were made based on the result of the pretest.

### Data collection procedure

The recruitment of study participants was carried out from February to November 2018. Women who fulfill the inclusion criteria were enrolled in the study during antenatal care. Participants were enrolled at 28–35 weeks of their gestational age. The follow-up period varies between participants depending on the time of enrollment to the study and the gestational age at enrollment. However, the overall follow up period was from February 2018 to February 2019. All selected women were followed by data collectors prospectively through pregnancy, delivery and the postnatal period to assess adverse perinatal outcomes of PIH. Data regarding maternal socio-demographic characteristics, medical and obstetric history using an interviewer-administered questionnaire and PIH status using medical records were collected during enrollment. At the second phase within 24 h of delivery, information about the adverse perinatal outcomes such as birth asphyxia, birth weight, and gestational age at delivery, stillbirth and early neonatal death were collected from medical records. During the third phase within the postnatal period of three to 7 days, data regarding early neonatal death and early neonatal admission to NICU were collected from medical records. The definition of outcomes was based on world health organization and other related literature [37–39]. Close supervision and checking of filled-in questionnaires were done by the field supervisors deployed with the data collectors. The overall data collection process was coordinated and supervised by the principal investigator.

### Definition of outcomes

The adverse perinatal outcome was defined as a newborn with the occurrence of any of the following outcomes low birth weight, birth asphyxia, small for gestational age, preterm delivery, admission to neonatal intensive care unit and perinatal death. Birth asphyxia was defined as a baby with trouble in breathing (gasping or breathing very irregularly or no breathing). Stillbirth was defined as a baby born with no signs of life at or after 28 weeks’ gestation [37]. Small for gestational age of pregnancy defined as a birth weight of newborn below the tenth percentile of weight distribution at the specified gestational age of a pregnancy [39]. Low birth weight was defined as a baby with a birth weight less than 2500 g [38]. Preterm delivery was defined as the delivery of the baby below 37 weeks gestation [38]. Low Apgar score defined as a newborn baby with an Apgar score of less than 7 at 1 and 5 min.

### Data analyses methods

Descriptive statistics, frequencies and percentage for categorical variable and summary statistics for continuous data (mean with standard deviation in normally distributed data or median with IQR if the data was not normally distributed) were used to characterize the study population. The normality distribution test was done using the Kolmogorov-Smirnov test and we considered as normally distributed if *p*-value > 0.05. Independent t-test was used to assess the mean difference between groups. The wealth index of participants was computed using principal component analysis (PCA) from socioeconomic variables. Due to the convergent problem in log-binomial regression analysis, a modified Poisson regression model with robust standard errors was used to calculate relative risk using STATA version 14 software (research resource identifiers (RRIDs: SCR_012763)) to identify the effect of PIH on adverse perinatal outcomes. Maternal age, wealth status, educational status, residence, gravidity, type of pregnancy (single or multiple births) and mode of delivery, anemia status, maternal undernutrition variables were controlled in the statistical models. After adjusted for confounders relative risk with 95% confidence interval and *p*-value < 0.05 was considered to declare statistical significance.

## Results

### Socio-demographic characteristics of study participants

**Fig. 1:**
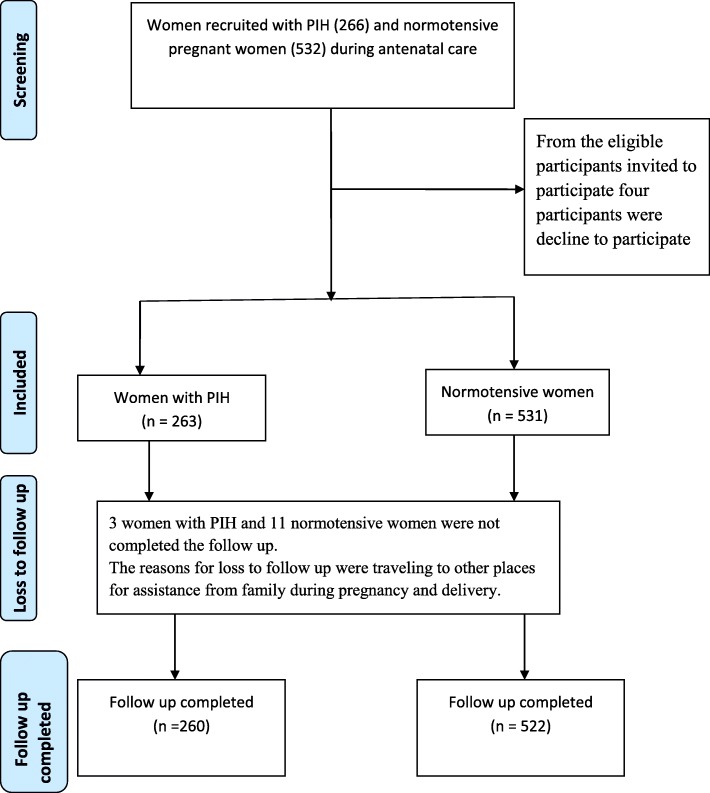
A flow diagram of study participants

Overall a total of 260(97.7%) women with PIH and 522(98.1%) normotensive pregnant women had completed the study (Fig. 1). The reasons for the loss-to-follow-up study were traveling to other places for assistance during pregnancy and delivery. The mean maternal age of women with PIH and normotensive women was more or less similar (27.27 vs 27.34 years respectively). Women with PIH were more likely residing in rural areas than normotensive women (31.5% vs 16.7%). The proportion of primigravidas were more likely higher among women with PIH than normotensive women (41.2% vs 31.8%). In addition, women PIH were less likely to be in the highest wealth status than normotensive women (20.1% vs 40.0%). The proportion of history of anemia was higher among women with PIH than normotensive women (27.3% vs 10%). (Table 1).

**Table 1:** Socio-demographic characteristics of pregnant women attending antenatal care in Tigray regional state, Ethiopia, 2019

Variable		Normotensive	Women with PIH
		Mean (SD)	Mean (SD)
Maternal Age (year)		27.34 (5.4)	27.27 (6.4)
		N(%)	N(%)
Residence	Urban	435 (83.3)	178 (68.5)
	Rural	87 (16.7)	82 (31.5)
Mothers educational status	No formal education	36 (6.9)	58 (22.3)
	Primary	114 (21.8)	103 (39.6)
	Secondary	188 (36.0)	62 (23.8)
	Diploma and above	184 (35.2)	37 (14.2)
Religious	Orthodox	459 (87.9)	214 (82.3)
	Catholic	17 (3.3)	15 (5.8)
	Muslim	38 (7.3)	26 (10.0)
	Protestant	8 (1.5)	5 (1.9)
Mothers occupation	Unemployed	250 (47.9)	201 (77.3)
	Employed	272 (52.1)	59 (22.7)
Wealth index	Lowest	120 (23.3)	138 (53.3)
	Middle	189 (36.7)	69 (26.6)
	Highest	206 (40.0)	52 (20.1)
Hospitals	Lemlem Carl	59 (11.3)	29 (11.2)
	Mekelle	78 (14.9)	39 (15)
	Wukro	50 (9.6)	24 (9.2)
	Adigrat	78 (14.9)	39 (15)
	Adwa	53 (10.2)	28 (10.8)
	Saint Marry Axum	79 (15.1)	39 (15)
	Suhul Shire	70 (13.4)	35 (13.5)
	Kahsay Abera	55 (10.5)	27 (10.4)
Gravidity	Primigravida	166 (31.8%)	107 (41.2%)
	2–4 gravida	299 (57.3%)	118 (45.4%)
	≥ 5 gravida	57 (10.9%)	35 (13.5%)
History of anemia	Yes	52 (10%)	71 (27.3%)
	No	470 (90%)	189 (72.7%)
Maternal undernutrition	Yes	119 (22.8%)	63 (24.2%)
	No	403 (77.2%)	197 (75.8%)
Type of pregnancy	Singleton	517 (99.0%)	245 (94.2%)
	Multiple	5 (1.0%)	15 (5.8%)
Prior history of PIH	Yes	9 (1.7%)	28 (12.7%)
	No	509 (98.3%)	192 (87.3%)
		Median (IQR)	Median (IQR)
Gestational age at enrollment (in weeks)		31 (±3.25)	31 (±4)

### Incidence of adverse perinatal outcomes among study participants

Nearly 66.4% of women with PIH and 22.2% of normotensive women developed adverse perinatal outcomes in their newborns. The incidence of birth asphyxia was higher among newborn babies delivered from women with PIH than normotensive women (46.5% vs11.3%). Similarly, low birth weight babies were higher among women with PIH than normotensive women (37.7% vs 6.1%). The mean birth weight of babies born from women with PIH was 2647.2 g and 3176 g among normotensive pregnant women (*t-test* = − 11.66, *p*-value *< 0.001*). In addition, 36.7% of babies born from women with PIH and 10.7% from normotensive women were small for gestational age. The proportions of admission to the neonatal intensive care unit were higher among newborns delivered from women with PIH than normotensive women (28.8% vs 5.4%). Additionally, higher preterm births occurred among pregnant women with PIH than normotensive pregnant women (40.8% vs 5.6%). Women with PIH were more likely to have a lower gestational age at delivery than normotensive women (37.3 ± 2.5 weeks vs. 39.0 ± 1.6 weeks, *p* = 0.001).

Stillbirths occurred in 10.0% of women with PIH and 1.7% normotensive pregnant women respectively. The stillbirth rate among women with PIH was 100/1000 live births.

**Fig. 2:**
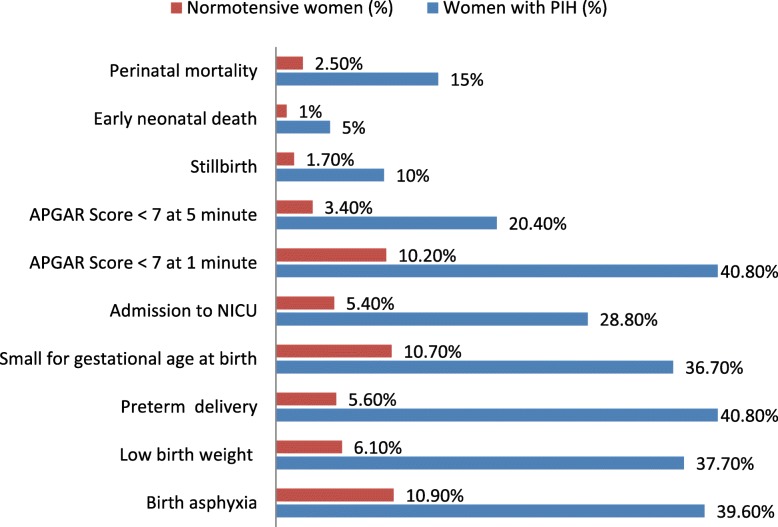
Adverse perinatal outcomes among women with PIH (*n* = 260) and normotensive pregnant women (*n* = 522) in Tigray Regional State, Ethiopia 2019

Moreover, early neonatal death occurred in 5.0% of mothers with PIH and 1.0% of normotensive mothers. Overall perinatal death occurred in 15.0% women with PIH and 2.5% normotensive pregnant women. Similarly, the perinatal mortality rate among women with PIH was 150 per 1000 live births and 25 per 1000 live births among normotensive pregnant women (Fig. 2).

### Risk of adverse perinatal outcomes associated with pregnancy-induced hypertension

The risk of birth asphyxia (adjusted relative risk (aRR) = 2.69, 95%CI (1.91,3.80)), low birth weight (aRR = 5.13 (3.36,7.84)), preterm newborn (aRR = 5.19(3.37,7.99)), small for gestational age (aRR = 3.3(2.3,4.6)), admission to NICU (aRR = 3.29(2.33,4.65)), stillbirth (aRR = 3.46(1.40,8.54)) and perinatal mortality (aRR = 3.67(1.83,7.38)) were significantly higher in women with pregnancy-induced hypertension compared to normotensive women after adjusted by maternal age, wealth status, educational status, residence, gravidity, mode of delivery, anemia and maternal undernutrition status (Table 2).

**Table 2:** Bivariate and multivariate Poisson regression analysis on the association between PIH and adverse perinatal outcomes in Tigray regional state, Ethiopia, 2018/2019

Perinatal outcome indicators		Pregnancy-induced hypertension *n* = 260	Normotensive women *n* = 522	Unadjusted RR (95% CI)	Adjusted RR (95% CI)
Birth asphyxia	Yes	103 (39.6%)	57 (10.9%)	3.62 (2.72,4.83)	2.69 (1.91,3.80)
	No	157 (60.4)	465 (89.1)	1	1
Low birth weight	Yes	98 (37.7%)	32 (6.1%)	6.14 (4.24, 8.90)	5.13 (3.36,7.84)
	No	162 (62.3)	490 (93.9)	1	1
Preterm delivery	Yes	106 (40.8%)	29 (5.6%)	7.33 (5.00,10.76)	5.19 (3.37,7.99)
	No	154 (59.2)	493 (94.4)	1	1
Small for gestational age at birth	Yes	95 (36.7%)	56 (10.7%)	3.41 (2.54,4.59)	3.29 (2.33,4.65)
	No	164 (63.3)	466 (89.3)	1	1
Admission to NICU	Yes	75 (28.8%)	28 (5.4%)	5.37 (3.57,8.08)	5.11 (3.10,8.40)
	No	185 (71.2)	494 (94.6)	1	1
Apgar Score < 7 at 1 min	Yes	106 (40.8%)	53 (10.2%)	4.01 (2.99,5.39)	2.93 (2.06,4.17)
	No	106 (40.8)	53 (10.2)	1	1
Apgar Score < 7at 5 min	Yes	53 (20.4%)	18 (3.4%)	5.91 (3.53,9.88)	3.78 (2.05,6.97)
	No	207 (79.6)	504 (96.6)	1	1
Stillbirth	Yes	26 (10.0%)	9 (1.7%)	5.79 (2.75,12.20)	3.46 (1.40,8.54)
	No	234 (90.0)	513 (98.3)	1	1
Early neonatal death	Yes	13 (5.0%)	5 (1.0%)	5.22 (1.87,14.49)	3.22 (1.06,9.74)
	No	247 (95.0)	517 (99.0)	1	1
Perinatal mortality	Yes	39 (15.0%)	13 (2.5%)	6.02 (3.27,11.08)	3.67 (1.83,7.38)
	No	221 (85.0)	509 (97.5)	1	

*RR* Relative Risk; ^*^the adverse perinatal outcomes were adjusted for maternal age, wealth status, educational status, residence, gravidity, type of pregnancy and mode of delivery, anemia status, maternal undernutrition 1 = reference group

## Discussion

The aim of this study was to assess the effect of pregnancy-induced hypertension on adverse perinatal outcomes. Based on this we found that women with PIH showed a higher risk of adverse perinatal outcomes such as low birth weight, birth asphyxia, small for gestational age, preterm delivery, stillbirth, admission to NICU and perinatal death compared to normotensive pregnant women.

The burden of PIH is common in Ethiopia. Many mothers and newborns are affected by the adverse outcomes of PIH. There are many causes for this problem such as poor access to health service and quality of maternal and newborn care in Ethiopia. Therefore, the rationale for this study was to help policymakers and programmers to have a clear picture about the effect of PIH on adverse perinatal outcomes to make an evidence-based decision and mobilize resources for the management of PIH and its associated perinatal complications in the region. It will also guide health care providers working in clinical areas to make evidence-based decisions for the prevention and management of adverse perinatal outcomes of PIH.

Specifically, findings of this study revealed that 37.7 and 40.8% of women with PIH delivered low birth weight (LBW) and preterm babies respectively. These findings were higher than the studies conducted in Ghana (24.7% LBW and 21.7% preterm), India (22.2% LBW and 24.6% preterm) and São Paulo city (21.0% LBW and 10.6% preterm) [40–42]. The difference in the incidence of low birth weight and preterm birth across studies could be due to the difference in the quality of antenatal care service and management of PIH between the study areas. Similarly, there was higher risk of delivering low birth weight and preterm newborn babies among women with PIH compared to normotensive women, this might be due to intrauterine growth retardation as a result of placental insufficiency and due to the interventional delivery being carried out irrespective of the gestational age, especially on eclamptics to prevent further maternal and perinatal morbidity and mortality. Complications from preterm birth and low birth weight are the leading cause of child deaths every year, accounting for nearly one million deaths globally. Thus, preventing and/or managing PIH should become as one of the priority ways of reducing the risk of low birth weight and preterm births as well as their associated consequences. Additionally, to improve the outcome of those premature newborn infants, health care providers should strengthen kangaroo mother care including thermal care (skin-to-skin contact), family support for the mother-infant, exclusive and frequent breastfeeding.

In addition, the incidence of birth asphyxia among newborn babies born from women with PIH was 39.6%. This finding was higher compared to studies conducted in Amhara Region, Ethiopia(10.1%), Ghana (15.2%), India (27.1%), Uganda (21.8%) and Turkey (28.9%) [24, 26, 40, 41, 43]. Additionally, the risk of birth asphyxia among newborns born from women with PIH was higher; this might be related to a decrease in the uteroplacental blood flow resulting from increased blood pressure [28]. Also, the risk of preterm birth of babies among women with PIH might be vulnerable to the immaturity of muscle tone and reflex irritability. In premature newborns, the lungs may be deficient in surfactant and this makes the lung more difficult to ventilate. Hence, neonatal resuscitation skilled health care providers should be assigned to every delivery service. In addition, all necessary types of equipment needed for resuscitation should be ready at every delivery by anticipating the risk of birth asphyxia among women with PIH since newborns that do not start breathing on their own by 1 min after birth should receive positive pressure ventilation with room air by a self-inflating bag and mask.

Further, the incidence of small for gestational age of newborns was 36.7% among women with PIH. This was higher than the study reports from Ghana (6.3%), Madagascar (25.7%), and South Africa (17%) [40, 44, 45]. The difference across studies might be related to the utilization of antenatal care services across countries. For instance, a study conducted in Ghana indicated that a higher proportion of pregnant women were completed four or more antenatal care visits compared to a study conducted in Ethiopia (41.3% vs 33.0%) [46, 47]. Moreover, the risk of small for gestational age newborns was higher among women with PIH; this could be related with intrauterine growth retardation due to a decrease of uteroplacental blood flow and the development of ischemia in women with pregnancy-induced hypertension. Thus, women with PIH should attend their delivery in facilities having neonatology skilled professional and necessary material for managing small for gestational age babies. Additionally, health care providers should provide immediate and adequate care for newborn infants with SGA to prevent further adverse effects of a newborn like hypoglycemia, hypothermia and feeding problems.

In this study, approximately one-third (28.8%) of the newborns delivered from women with PIH were admitted to the neonatal intensive care unit (NICU). This finding was higher than studies conducted in Iran (13%) and Minia maternity hospital, Egypt (18.8%) [30, 48]. The higher risk of newborn baby admission to NICU among newborns delivered from women with PIH compared to newborn delivered from normotensive women could be related to the higher adverse effect of PIH on low birth weight, birth asphyxia, and preterm births.

The incidence of stillbirth among women with PIH in this study was 10%. This result was more or less consistent with the study conducted in Mizan Tepi, Ethiopia (9.1%) and Mettu, Ethiopia (10%) [27, 49], but the finding of this study was higher than the study conducted in Zimbabwe (5.4%), Ghana (6.8%) [23, 40]. This difference could be due to the difference in the quality of antenatal care and obstetric care service among health care institutions. The increased risk of stillbirth among women with PIH might be related with the effect of decreased uteroplacental blood flow and placental ischemia related to PIH which compromises blood flow to the fetus [50, 51].

Similarly, the result of this study revealed that perinatal death occurred in 15.0% of women with PIH. This finding was more or less consistent with the study conducted in Mettu Karl Referral Hospital, Ethiopia (12.04%) [27]. However, the finding of this study was higher than a study conducted in Ghana (10.6%), Port-Harcourt, Nigerian (7.6%), Madagascar (8.7%) and University college hospital, Ibadan (10%) [40, 52, 53]. The difference in the incidence of perinatal death across studies could be due to the difference in the quality of care for antenatal care, intrapartum and newborn care among health care facilities. This finding is far higher from the targeted global plan of sustainable development goal (SDG) to reduce neonatal mortality to less than 12 per 1000 live births and national health sector transformation plan to reduced 15/1000 live births [54, 55]. This indicates the necessity of Ethiopia federal ministry of health and Tigray regional health bureau to strengthen maternal and newborn health care in order to achieve the targeted global and national SDG plan by focusing interventions on the determinants of perinatal mortality such as pregnancy-induced hypertension.

The prospective nature of the study considered as a strength of this study in reducing the incompleteness of data and less bias in misclassification due to the prospective evaluation of exposures and outcomes. We have enrolled the participants at 28–35 weeks of gestational age. The reasons why we did not include women who developed PIH before 28 weeks in this study were; i) the number of women who develop PIH before 28 weeks is likely to be very small [10, 56]; ii) those women who develop PIH early could be more likely to have more complications and this might overestimate the rates of complications in the PIH group [56, 57].

Similarly, the reasons to exclude the pregnant women after 36 weeks was; i) to see all adverse perinatal outcomes over a long period of time before the mother gives birth, ii) another reason was also due to the management protocols of PIH, after 36 weeks of gestational age obstetrician could manage with elective/emergency cesarean section [58], so this condition may underestimate the adverse perinatal outcomes especially stillbirth.

As a limitation, matching criteria were not used to identify normotensive women this could reduce the comparability of the outcomes, the tertiary hospital in Tigray regional state was not included in this study; this might have some effect on the burden of adverse perinatal outcomes of PIH.

## Conclusions

Higher incidences of adverse perinatal outcomes occurred among women with pregnancy-induced hypertension in Tigray regional state, Ethiopia. Pregnancy-induced hypertension was associated with a higher risk of adverse perinatal outcomes such as low birth weight, birth asphyxia, small for gestational age, preterm delivery and perinatal death. Tigray Regional Health Bureau and district health offices should use this evidence to improve perinatal health outcomes in collaboration with other stakeholders. In addition, health care providers should strengthen the primary and secondary prevention, early diagnosis and prompt management of pregnancy-induced hypertension to reduce the incidence of adverse perinatal outcomes of pregnancy-induced hypertension. This study addresses women diagnosed with preeclampsia, eclampsia and gestational hypertension between 28 and 35 weeks of gestation. Hence, we recommended researchers to conducted further study about the effect of early (before 28 wks) and late (after 36 wks) pregnancy-induced hypertension on the adverse fetal outcomes.

## Data Availability

The datasets used and/or analyzed during this study are available from the corresponding author on reasonable request.

## Abbreviation

Apgar score: Appearance, Pulse, Grimace, Activity, and Respiration score
APH: Antepartum hemorrhage
aRR: Adjusted Relative Risk
BP: Blood Pressure
EDHS: Ethiopia Demographic Health Survey
LBW: Low birth weight
NICU: Neonatal Intensive Care Unit
PIH: Pregnancy Induced Hypertension
RR: Crude relative risk
SGA: Small for gestational age
WHO: World Health Organization
